# Analysis of *RIOK2* Functions in Mediating the Toxic Effects of Deoxynivalenol in Porcine Intestinal Epithelial Cells

**DOI:** 10.3390/ijms232112712

**Published:** 2022-10-22

**Authors:** Zhongcheng Gao, Chao Xu, Hairui Fan, Haifei Wang, Zhengchang Wu, Shenglong Wu, Wenbin Bao

**Affiliations:** 1 Key Laboratory for Animal Genetics, Breeding, Reproduction and Molecular Design, College of Animal Science and Technology, Yangzhou University, Yangzhou 225009, China; 2 Joint International Research Laboratory of Agriculture & Agri-Product Safety, Yangzhou University, Yangzhou 225009, China

**Keywords:** pigs, *RIOK2*, deoxynivalenol, cytotoxicity, Sp1, MAPK

## Abstract

Deoxynivalenol (DON) is a type of mycotoxin that threatens human and livestock health. Right open reading frame kinase 2 (RIOK2) is a kinase that has a pivotal function in ribosome maturation and cell cycle progression. This study aims to clarify the role of the *RIOK2* gene in DON-induced cytotoxicity regulation in porcine intestinal epithelial cells (IPEC-J2). Cell viability assay and flow cytometry showed that the knockdown of *RIOK2* inhibited proliferation and induced apoptosis, cell cycle arrest, and oxidative stress in DON-induced IPEC-J2. Then, transcriptome profiling identified candidate genes and pathways that closely interacted with both DON cytotoxicity regulation and *RIOK2* expression. Furthermore, *RIOK2* interference promoted the activation of the MAPK signaling pathway by increasing the phosphorylation of ERK and JNK. Additionally, we performed the dual-luciferase reporter and ChIP assays to elucidate that the expression of *RIOK2* was influenced by the binding of transcription factor Sp1 with the promoter region. Briefly, the reduced expression of the *RIOK2* gene exacerbates the cytotoxic effects induced by DON in IPEC-J2. Our findings provide insights into the control strategies for DON contamination by identifying functional genes and effective molecular markers.

## 1. Introduction

Mycotoxins are harmful secondary metabolites produced by fungi or molds under certain conditions. More than 300 mycotoxins cause considerable losses in human health and animal husbandry annually [1]. Deoxynivalenol (DON), one of the most prevalent mycotoxins in food and feedstuff, is a type B trichothecene derived from *Fusarium* species [2,3]. The ingestion of feed containing DON can lead to vomiting, anorexia, diarrhea, or even death, posing a severe threat to the livestock industry [4]. Pigs are the most sensitive animals to mycotoxins [5]. Even trace amounts of DON can cause damage to intestinal health and impaired immune function [6]. At the molecular level, DON inhibits the synthesis of protein by binding to the ribosome and disrupts proliferation, differentiation, and apoptosis by activating signal transduction kinases [7,8]. Numerous treatments for DON degradation, including thermal, chemical, biological, and atmospheric cold plasma treatments, have been certified to have certain limitations [9]. Therefore, screening functional genes and molecular markers may be a practical approach to regulating the cytotoxic effects induced by DON.

The right open reading frame kinases (RIOKs), a family of atypical protein kinases, including RIOK1, RIOK2, and RIOK3, are present in all eukaryotes [10]. RIOK2 (also known as RIO2), a member of the RIOKs, is a serine/threonine kinase that performs a critical role in ribosome maturation and cell cycle progression [11,12,13]. RIOK2 was identified as an indispensable trans-acting factor with an ATPase-dependent function during the maturation of the 40S subunit [11,14]. It has been observed that *RIOK2* was highly expressed in malignant tumors with vital functions [15,16]. Our previous study demonstrated a significant increase in the expression of *RIOK2* by performing transcriptome analysis of DON-treated IPEC-J2 [17], suggesting that it might play an essential physiological role during DON exposure. Nevertheless, the potential functions and regulatory mechanisms of the *RIOK2* gene in DON-induced IPEC-J2 remain to be elucidated.

In this study, the porcine intestinal epithelial cell line was used as a model to investigate the biological functions of *RIOK2* during DON exposure via small interfering RNAs (siRNAs). Firstly, cell viability and flow cytometry assays were performed to determine the effects of *RIOK2* in cell proliferation, apoptosis, cell cycle, and oxidative stress. Furthermore, transcriptome sequencing revealed the genes downstream of *RIOK2* involved in related pathways or networks in DON-induced IPEC-J2. Finally, we investigated the effect of transcription factor Sp1 on the *RIOK2* gene expression to clarify the underlying molecular regulatory mechanisms. This study not only revealed the biological functions of the *RIOK2* gene on DON-induced cytotoxicity in IPEC-J2, but also provided a theoretical basis for identifying molecular markers related to DON detoxication.

## 2. Results

### 2.1. Down-Regulation of RIOK2 Increased Oxidative Stress Induced by DON

**Figure 1:**
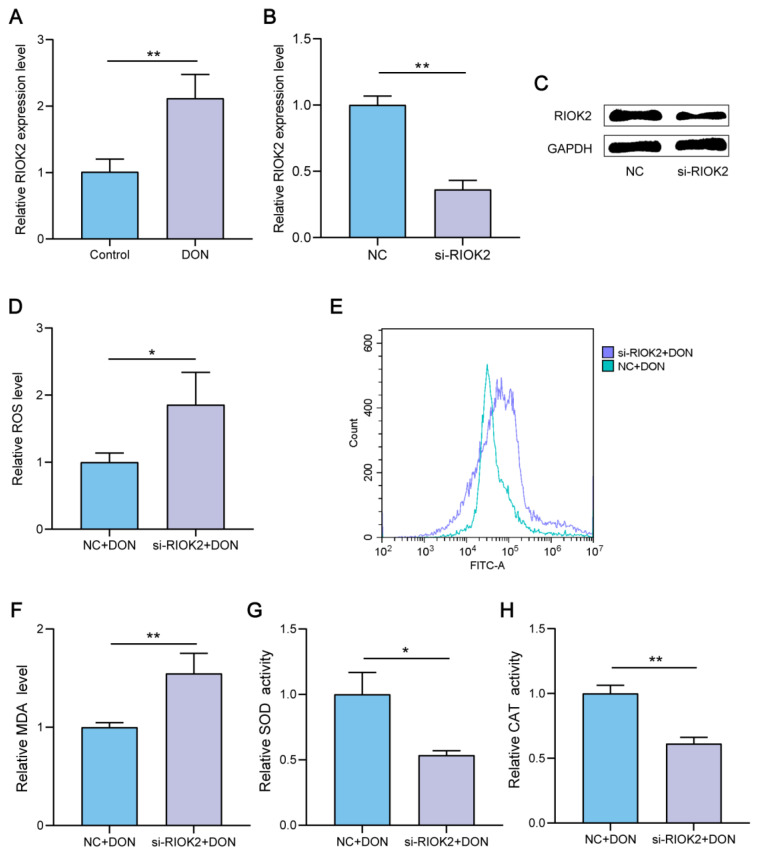
Effects of silencing *RIOK2* on oxidative stress markers in DON-induced IPEC-J2. (**A**) *RIOK2* mRNA expression levels in DON-induced cells and control cells. (**B**,**C**) Interference efficiency detection after transfection with siRNAs. (**D**,**E**) Fluorescence intensities in IPEC-J2 detected by flow cytometry. (**F**–**H**) MDA level, SOD activity, and CAT activity determined by assay kits. IPEC-J2 were transfected with si-*RIOK2* or negative control and treated with DON for 48 h. Each experiment was replicated at least three times, and data are presented as mean values ± SD. * *p* < 0.05, ** *p* < 0.01.

To explore the relationship between *RIOK2* expression and DON exposure, we used qPCR to detect the expression level of the *RIOK2* gene. The results showed that the *RIOK2* expression in DON-treated cells was significantly upregulated (Figure 1A). To verify the role of the *RIOK2* gene during DON exposure, we downregulated the *RIOK2* expression through RNA interference. Both qPCR and Western blotting analysis showed that the *RIOK2* expression was significantly downregulated compared with the negative control group (Figure 1B,C). DON causes the excessive generation of reactive oxygen species (ROS), which leads to intracellular oxidative stress and cell damage [18,19]. According to the analysis of the relative fluorescence intensity, the interference of *RIOK2* increased the level of ROS (Figure 1D,E). To further evaluate the role of *RIOK2* in antioxidation after DON exposure, we measured the MDA level, SOD activity, and CAT activity. Data revealed that the knockdown of *RIOK2* increased the level of MDA (Figure 1F) and suppressed the activity of SOD (Figure 1G) and CAT (Figure 1H).

### 2.2. Effects of RIOK2 on Cell Viability, Apoptosis, and Cell Cycle with DON Treatment

**Figure 2:**
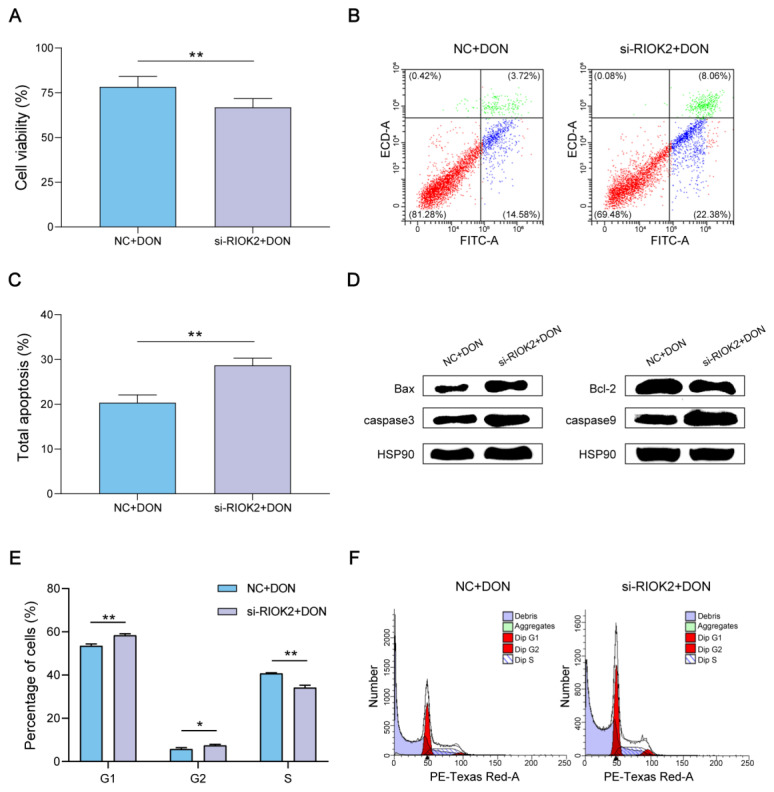
Silencing *RIOK2* induced proliferation inhibition, apoptosis, and cell cycle arrest in DON-induced IPEC-J2. (**A**) The proliferation ability analysis of IPEC-J2 using the cell viablity assay. (**B**,**C**) Apoptotic assay by flow cytometry. Apoptotic cells were Annexin V-positive and PI (propidium iodide)-negative. (**D**) Western blotting analysis of the changes in the protein levels of related apoptosis factors (Bax, Bcl-2, caspase3, and caspase9). (**E**,**F**) Analysis of percentage of IPEC-J2 using flow cytometry. IPEC-J2 were transfected with si-*RIOK2* or the negative control, and were treated with DON for 48 h. Each experiment was replicated at least three times, and data are presented as mean values ± SD. * *p* < 0.05, ** *p* < 0.01.

DON disrupts proliferation, differentiation, and apoptosis by acting on the ribosome [20]. The cell viability assay showed that the knockdown of *RIOK2* significantly decreased the viability of IPEC-J2 (Figure 2A). Flow cytometry suggested that knockdown of *RIOK2* considerably induced apoptosis in IPEC-J2 (Figure 2B,C), accompanied with increases in the pro-apoptotic proteins (Bax, caspase3, and caspase9) and decreases in the anti-apoptotic protein Bcl-2 in IPEC-J2 (Figure 2D). Finally, we analyzed the percentage of cells, and the data indicated that interference of *RIOK2* increased the G1 and G2 phases and decreased the S phase in DON-induced IPEC-J2 (Figure 2E,F).

### 2.3. Transcriptome Sequencing Screening and Validation of Genes and Pathways Associated with RIOK2 upon DON Exposure

**Figure 3:**
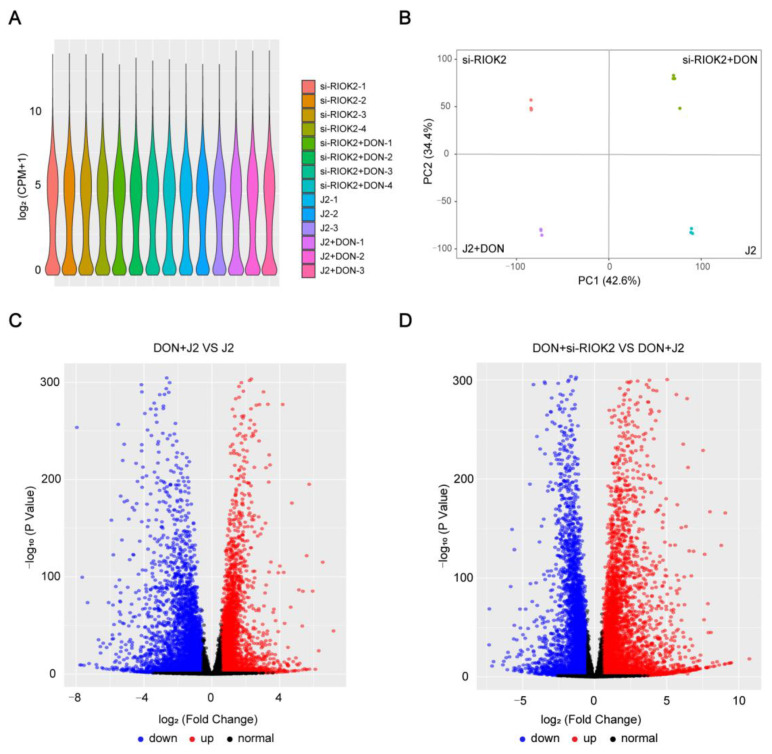
Data quality control of RNA-seq and preliminary analysis. (**A**) Violin box plot of CPM distribution among all the samples. (**B**) Principal component analysis of the four groups of samples. PC1 means 42.6 of the variance, and PC2 means 34.4% of the variance. (**C**,**D**) Volcano plots of the differentially expressed genes. The blue, red, and black nodes represent downregulated, upregulated, and normal genes, respectively.

To examine the molecular mechanisms underpinning DON resistance of the *RIOK2* gene in IPEC-J2, we performed the systemic analysis at the transcriptome level. The violin plot distribution of counts per million (CPM) showed the degree of dispersion in gene abundance distribution (Figure 3A). Principal component analysis (PCA) showed complete separation between all four groups of samples (Figure 3B). As shown in the volcano plots, 1828 genes were upregulated, and 2837 genes were downregulated in the group DON + J2 VS J2 (Figure 3C). Another comparison between the DON + si-*RIOK2* group and the DON + J2 group identified 3200 upregulated genes and 2736 downregulated genes (Figure 3D). The differentially expressed genes are shown in Tables S1 and S2 for the pairwise comparisons between different treatments.

**Figure 4:**
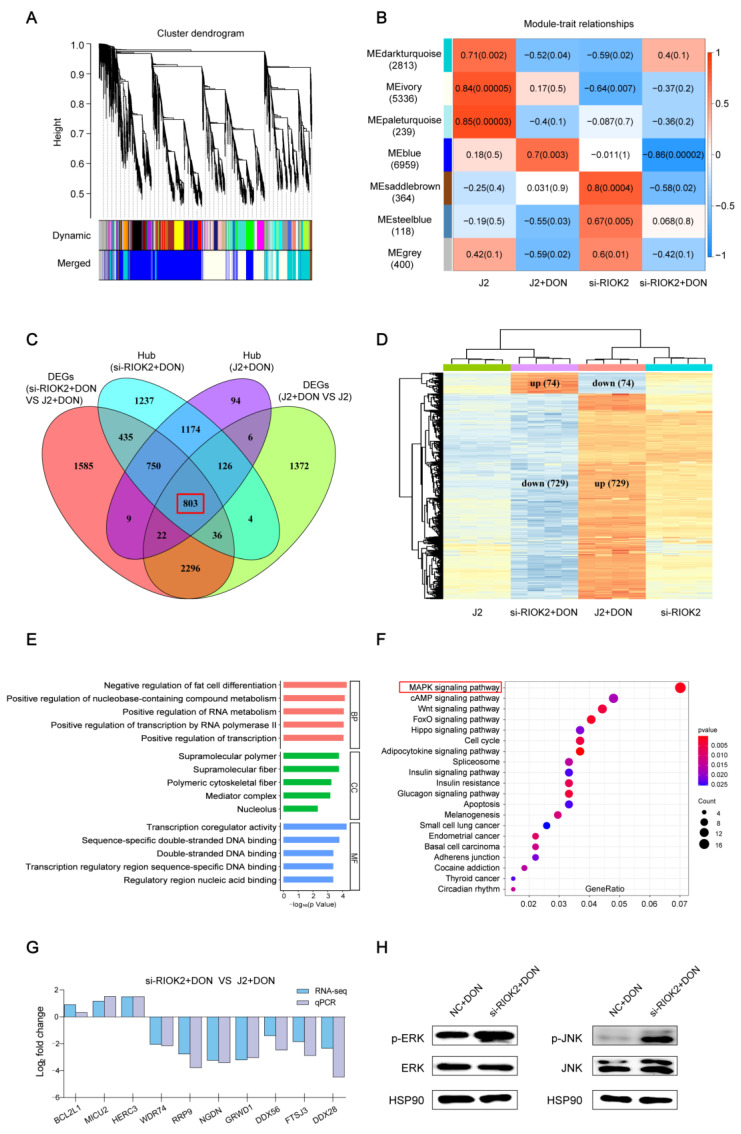
WGCNA showing the mechanism of the downstream regulation of the *RIOK2* gene. (**A**) WGCNA cluster dendrogram and module assignment. Colors in horizontal bars indicate modules, and branches refer to highly connected gene clusters. (**B**) Heatmap of the correlation between the module eigengenes and different treatment groups. Each cell contains the correlation coefficient and *p*-value. (**C**) Overlapping differentially expressed genes and WGCNA blue module hub genes uncover key hub DEGs in DON-induced cytotoxicity regulation. (**D**) Cluster analysis graph of differential gene samples between different groups. (**E**) GO enrichment analysis of 803 hub genes. (**F**) KEGG pathway analysis of 803 hub genes. The significance of enrichment gradually increases from blue to red, and the size of the dots indicates the number of genes contained in the corresponding pathway. (**G**) qPCR validation of differentially expressed genes. (**H**) Inhibition of *RIOK2* promotes the activation of the MAPK signaling pathway in DON-induced IPEC-J2.

To accurately identify hubs of gene clusters associated with the *RIOK2* gene that function in the cytotoxic effects induced by DON, we analyzed all four groups of genes by the WGCNA method. All of the genes were clustered into different modules based on their correlation. A total of seven modules were identified and labeled with a unique color (Figure 4A). The blue module was significantly correlated with DON-induced cells, either the positive correlation with the DON-treated IPEC-J2 (J2 + DON) or the negative correlation with the DON-treated *RIOK2* gene interference cell line (si-*RIOK2* + DON) (Figure 4B). By refining the genes within the blue module of J2 + DON and si-*RIOK2* + DON groups, the module membership cut-off of ≥0.8 and the gene significance cut-off of ≥0.6 identified 2984 and 4565 hub genes, respectively. To identify overlapping hub genes that may be especially significant in the cytotoxic effects induced by DON, we selected 803 genes (Table S3) from two traits of the blue module identified from the WGCNA analysis (J2 + DON (Table S4) and si-*RIOK2* + DON (Table S5)), which differed significantly among two groups of DEGs (J2 + DON VS J2 and si-*RIOK2* + DON VS J2 + DON) (Figure 4C). Then, 803 genes divided into four groups were shown in the heatmap. Interestingly, 729 genes were upregulated in the group of J2 + DON and downregulated in the group of si-*RIOK2* + DON. The remaining 74 genes also showed the opposite expression trends in these two groups (Figure 4D). Gene Ontology (GO) enrichment analysis (Table S6) showed that 803 genes were associated with the cell differentiation, RNA metabolic, and nucleic acid-templated transcription processes (Figure 4E). The Kyoto Encyclopedia of Genes and Genomes (KEGG) pathway enrichment analysis (Table S7) showed that these genes might be mainly involved in the mitogen-activated protein kinases (MAPK) signaling pathway, cAMP signaling pathway, and Wnt signaling pathway (Figure 4F). Ten differentially expressed genes (*BCL2L1*, *MICU2*, *HERC3*, *WDR74*, *RRP9*, *NGDN*, *GRWD1*, *DDX58*, *FTSJ3,* and *DDX28*) were randomly selected for qPCR validation. As expected, the expression trends of the qPCR results were consistent with the transcriptome sequencing results (Figure 4G). Based on the KEGG pathway enrichment analysis, the most significant difference was in the MAPK signaling pathway. There may be a probable link between the activation of the MAPK pathway and the interference of the *RIOK2* gene. As a result, the knockdown of *RIOK2* effectively increased the phosphorylation of extracellular signal-regulated kinase (ERK) and c-Jun N-terminal kinase (JNK) in DON-induced IPEC-J2, which means the MAPK pathway was activated (Figure 4H).

### 2.4. Effects of Transcription Factor Sp1 on RIOK2 Expression

**Figure 5:**
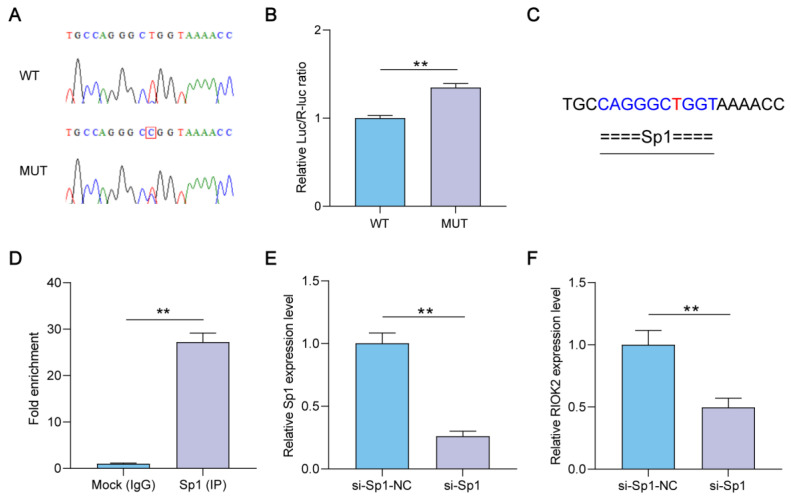
Sp1 activates the *RIOK2* expression. (**A**) Sequencing plots of SNPs of the *RIOK2* gene. WT means wild-type sequence and MUT means mutant sequence. The C/T mutation site is marked with a red box. (**B**) The dual-luciferase reporter gene assay was used to evaluate the effect of the mutation site on the *RIOK2* promoter activity. (**C**) Prediction of Sp1 potential binding sites. The mutation site is highlighted in red. (**D**) ChIP-qPCR demonstrated that Sp1 could bind the promoter of *RIOK2*. (**E**) Detection of *Sp1* gene interference efficiency by qPCR. (**F**) The expression of the *RIOK2* gene in IPEC-J2 transfected with si-*Sp1*. Each experiment was replicated at least three times, and data are presented as mean values ± SD. ** *p* < 0.01.

To explore the potential molecular markers associated with DON resistance in pigs, we amplified the promoter sequence of the *RIOK2* gene to identify the single nucleotide polymorphism (SNP) sites. Sequencing of the PCR products detected one mutation (C/T) located at 663 bp upstream of the *RIOK2* gene in the Large White pigs (Figure 5A). Wild-type and mutant vectors in the *RIOK2* promoter region were transfected into IPEC-J2, and the luciferase activity was measured. The relative luciferase activity of the mutation vector was significantly higher than that of the wild-type vector (Figure 5B). The prediction result indicated that the mutation site was located within the binding sites of transcription factor Sp1 (Figure 5C). We assumed that Sp1 could directly bind to the promoter area of the *RIOK2* gene in IPEC-J2. As a result, the analysis of the ChIP-qPCR assay verified our hypothesis (Figure 5D). To further explore the regulation of Sp1 on *RIOK2* expression, we constructed the siRNA interference vector of the *Sp1* gene. qPCR showed a significantly lower expression of the interfering vector than the negative control (Figure 5E). Interestingly, *Sp1* silencing significantly suppressed the expression level of the *RIOK2* gene, indicating that the Sp1 transcription factor plays a vital role in regulating *RIOK2* gene expression in IPEC-J2 (Figure 5F).

## 3. Discussion

The emergence of DON in feed is an inevitable and severe problem in the animal husbandry worldwide [21]. Moreover, DON also severely threatens human health through ecological cycles [22]. Thus, many physical and chemical strategies for DON decontamination in feed have been developed. However, few meet the requirements for practical applications due to efficiency, safety, or cost [23,24,25]. Nowadays, biological control has gradually attracted scientists’ attention as a novel promising method [26]. Peroxidase and aldo-keto reductase have shown significant detoxification of DON owing to the mechanism of degradation or transformation [27,28]. Nevertheless, few studies on functional genes associated with DON detoxification and their molecular mechanisms have been reported. At the molecular level, DON targets the ribosome by binding to the A position of the peptidyl transferase center (PTC) of cells [29,30]. The interaction of DON with ribosomes leads to the prolongation of the chain extension step of protein synthesis and inhibits the synthesis of DNA, RNA, and proteins [7]. Our previous study identified a subset of genes relevant to protein degradation and ribosome synthesis by transcriptome analysis, in which *RIOK2* showed significant expression changes upon DON exposure [17]. Thus, we hypothesized that the *RIOK2* gene performs critical biological functions in response to DON exposure in IPEC-J2.

Previous studies have shown that a reduced expression of *RIOK2* caused proliferation inhibition, apoptosis, and cell cycle arrest in *Drosophila* glioblastoma cells and human leukemic cells [31,32]. Herein, we also illustrated that in DON-induced IPEC-J2, the knockdown of *RIOK2* exacerbated the cytotoxic effects in proliferation, cell cycle, and apoptosis. Among them, the proportional changes in each phase of the cell cycle visually shows the decisive effect of *RIOK2* on protein biosynthesis. Significant decreases in the proportion of the DNA synthesis phase were observed, accompanied by increases in the G1 phase, representing pre-DNA synthesis. Oxidative stress, caused by a mismatch between free radical production and cellular defense ability, has been confirmed as one of the cytotoxic effects induced by DON in existing studies [33,34,35]. It has been reported that *RIOK1* was correlated with the transcription factor SKN-1, which is critical for oxidative stress resistance [36,37]. Thus, we performed flow cytometry to determine the reactive oxygen species level for illustrating the link between *RIOK2* expression and cellular resistance to oxidative stress induced by DON. The results suggested that *RIOK2* expression is closely correlated with the ROS level, one of the indicators of oxidative stress. In addition, the consequences of oxidant factor MDA and antioxidant enzymes SOD, as well as CAT, also supported this point of view. Briefly, we proved that the reduced expression of *RIOK2* exacerbated the phenotypic cytotoxic effects of DON on IPEC-J2, including cell viability, apoptosis, cell cycle, and oxidative stress. Considering the significant upregulation of *RIOK2* expression in DON-induced cells, this might be one of the response mechanisms through which IPEC-J2 resist the toxicity caused by DON.

Activation of MAPKs, mitochondrial signaling pathways, and inhibition of protein synthesis are the main mechanisms of DON-induced immunotoxicity [38]. Notably, the results of the transcriptome analysis showed that the MAPK signaling pathway was remarkably enriched. So, we speculated that it might play a crucial role via interacting with *RIOK2* for responses to DON exposure. A recent study suggested that RIOK2 is a new target of the MAPK-activated kinase RSK [39]. As components of the MAPK signaling pathway, ERK and JNK are associated with the regulation of cell survival and death [40,41]. In this study, knockdown of *RIOK2* also significantly increased the phosphorylation of ERK and JNK, representing the activation of MAPKs in DON-induced IPEC-J2. It has been confirmed that DON induces ERK and JNK migration to the 40S ribosomal subunit and their phosphorylation [42]. In consideration of the critical effect of *RIOK2* on the 40S maturation, it is possible that the increased expression of *RIOK2* might attenuate DON-induced cytotoxicity by targeting the 40S subunit in a MAPK-dependent manner.

**Figure 6:**
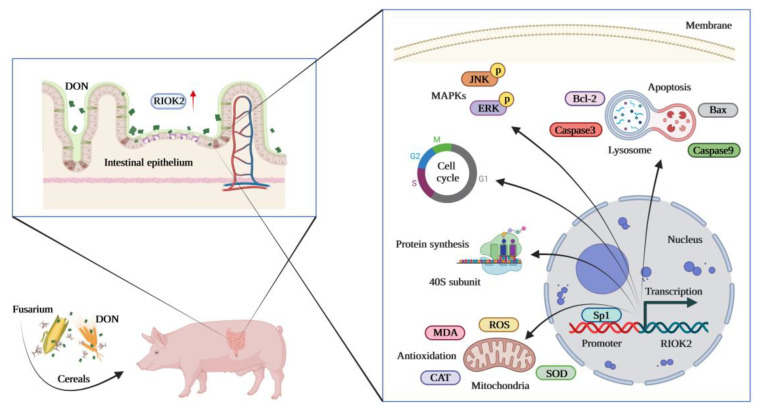
Schematic diagram of the *RIOK2* gene regulating the cytotoxic effects induced by DON in IPEC-J2. DON enters the small intestines of pigs by ingesting cereals infected with *Fusarium*. *RIOK2* is highly expressed during DON exposure and improves cytotoxicity resistance by affecting cell proliferation, apoptosis, antioxidant capacity, and activation of the MAPK signaling pathway in IPEC-J2. Moreover, the transcription factor Sp1 regulates the expression of *RIOK2* by binding to the promoter region. This mechanism determines the action of *RIOK2* in the responses of IPEC-J2 to DON exposure. Created with BioRender.com.

In this study, we uncovered the crucial role of *RIOK2* in mediating the toxic effects of DON in IPEC-J2. Furthermore, relevant pathways and differentially expressed genes related to *RIOK2* involved in DON cytotoxicity regulation were found to elucidate the specific molecular mechanisms during this dynamic equilibrium process. Activation of the MAPK signaling pathway may be a potential cause of exacerbated DON-induced cytotoxicity in IPEC-J2 due to *RIOK2* knockdown. Additionally, the binding of the transcription factor Sp1 with the promoter region could regulate the expression of *RIOK2*, which might indirectly affect the protection of IPEC-J2 from DON damage (Figure 6). To summarize, our findings further the understanding of the potential connection between *RIOK2* and transcription regulator Sp1 for DON tolerance in IPEC-J2, and provide a theoretical basis for identifying molecular markers associated with DON cytotoxicity, as well as the prevention and control strategies for DON contamination.

## 4. Materials and Methods

### 4.1. Cell Culture and DON Treatment

The incubation of IPEC-J2 at 37 °C in 5% CO_2_ with Dulbecco’s Modified Eagle Medium (DMEM) containing 10% fetal bovine serum (FBS) (Gibco., Grand Island, NY, USA) was conducted using cells provided by the University of Pennsylvania (Philadelphia, PA, USA). The cells were treated with 1 μg/mL DON (C_15_H_20_O_6_; Sigma-Aldrich, St. Louis, MO, USA) for 48 h, as described in our previous study [17].

### 4.2. RNA Interference

All of the siRNA sequences (si-*RIOK2*, si-*RIOK2*-NC, si-*Sp1*, and si-*Sp1*-NC) listed in Table S8 were designed and synthesized by Gene Pharma Co., Ltd. (Suzhou, China). Each treatment group was treated with three replicates of each vector using Lipofectamine 3000 (Invitrogen Co., Ltd., Carlsbad, CA, USA). After incubation for 48 h, the cells were collected for interference efficiency detection by qPCR and Western blotting analysis.

### 4.3. qPCR Analysis

RNAiso (Takara, Dalian, China) was used to extract the total RNA from IPEC-J2. Then, the RNA was reversely transcribed into cDNA using a cDNA synthesis kit (Vazyme Biotech Co., Ltd., Nanjing, China). Based on the sequences published in the GenBank database and Primer Premier 5.0 software, the primers (Table S9) were designed and synthesized by Sangon Biotech (Shanghai, China). The reaction was performed using an ABI 7500 Fast Real-Time Quantitative PCR System (Applied Biosystems, Foster City, CA, USA) with a 20 μL mixture containing 2 μL of cDNA template, 10 μL of 2 × AceQ Universal SYBR qPCR Master Mix, 0.4 μL each of forward and reverse primers, and 7.2 μL of ddH_2_O. The reaction was conducted at 95 °C for 5 min, followed by 40 cycles of 95 °C for 10 s and 60 °C for 30 s. GAPDH was chosen as an internal control, and the relative gene expression was calculated using the 2^−∆∆Ct^ method [43].

### 4.4. Western Blotting Analysis

The cells were washed with PBS and lysed in a radioimmunoprecipitation assay (RIPA) buffer containing proteinase inhibitor and phosphatase inhibitor on ice for 20 min. After centrifugation, the supernatant was gathered to determine the protein concentration using a BCA Protein Assay Kit (Beyotime, Shanghai, China). After dilution with a 5 × SDS-PAGE buffer, the protein samples were denatured at 95 °C for 10 min. Then, the proteins were separated in sodium dodecyl sulfate–polyacrylamide gel electrophoresis (SDS-PAGE) and transferred to the polyvinylidene fluoride (PVDF) (Millipore, Shanghai, China) membrane. The membranes were blocked with 5% skimmed milk for 2 h and incubated with primary antibodies [anti-RIOK2 (Abclonal, Woburn, MA, USA, A12122), anti-Bax (HuaBio, Woburn, MA, USA, ET1603-34), anti-Bcl-2 (HuaBio, ET1603-11), anti-caspase3 (HuaBio, ER30804), anti-caspase9 (HuaBio, ET1603-27), anti-phospho-ERK (Abcam Ltd., Cambridge, UK, ab201015), anti-ERK (Abcam, ab109282), anti-phospho-JNK (Abcam, ab124956), and anti-JNK (Abcam, ab179461)) at 4 °C overnight. After being washed thrice with TBST, the membranes were incubated with goat anti-rabbit IgG antibody (Abcam, ab205718) or anti-mouse IgG antibody (HuaBio, HA1006) at room temperature for 1.5 h. Finally, they were visualized with an Enhanced Chemiluminescent Detection Kit (ThermoFisher Scientific, Waltham, MA, USA) and exposed to the FluorChem FC3 system (Protein-Simple, San Jose, CA, USA). GAPDH (Proteintech Ltd., Rosemont, IL, USA, 10494-1-AP) or HSP90 (Proteintech Ltd., 60318) was used as a loading control.

### 4.5. Assessment of Cell Viability

Cell viability was measured using a Cell Viability Assay Kit (Yeasen Biotechnology Co., Ltd., Shanghai, China), according to the manufacturer’s instructions. The absorbance was measured at a wavelength of 450 nm using a microplate reader (Sunrise, Tecan, Switzerland).

### 4.6. Cell Apoptosis Assay

The Annexin V-fluorescein isothiocyanate (FITC)/propidium iodide (PI) Apoptosis Detection Kit (Beijing Solarbio Science and Technology Co., Ltd., Beijing, China) was used to detect the cell apoptosis. In accordance with the manufacturer’s instructions, centrifugally collected cells were washed and stained with Annexin V-FITC and propidium iodide (PI). Data were acquired by CytExpert Flow Cytometer (Beckman Coulter, Brea, CA, USA) and analyzed by CytExpert 2.3 (Beckman Coulter, Brea, CA, USA).

### 4.7. Cell Cycle Analysis

According to a Cell Cycle and Apoptosis Analysis Kit (Beyotime Institute of Biotechnology, Haimen, China), the cells were fixed in 70% ice alcohol at 4 °C overnight and were stained with propidium iodide (PI) according to the manufacturer’s protocol. The percentage of cells in different phases was analyzed using ModFit LT 3.0 (Verity Software House, Topsham, ME, USA).

### 4.8. Measurement of Reactive Oxygen Species (ROS)

To determine the effects of the downregulation of *RIOK2*, we measured the ROS levels with a Reactive Oxygen Species Assay Kit (Beijing Solarbio Science and Technology Co., Ltd., Beijing, China). After being washed thrice with PBS, the cells were incubated with serum-free DMEM containing 2′,7′-dichlorofluorescein diacetate (DCFH-DA) (10 μM) for 20 min. Finally, the ROS level was detected using a CytExpert Flow Cytometer (Beckman Coulter, Brea, CA, USA) with a 488 nm excited wavelength and analyzed by CytExpert 2.3 (Beckman Coulter, Brea, CA, USA).

### 4.9. Detection of Oxidative Stress Markers

The cells with PBS were homogenized on ice and centrifuged to obtain the supernatants. The malondialdehyde (MDA) level, superoxide dismutase (SOD) activity, and catalase (CAT) activity were separately determined using the MDA assay kit, SOD assay kit, and CAT assay kit (Nanjing Jiancheng Bioengineering Institute, Nanjing, China), according to the manufacturer’s protocols.

### 4.10. Dual-Luciferase Reporter Assays

The cells were collected 48 h post-transfection, according to the Dual-Luciferase Reporter Assay Kit (Vazyme Biotech Co., Ltd., Nanjing, China). The activity of Firefly luciferase (Ff) and Renilla luciferase (Rn) was measured using a Tecan Infinite 200 microplate reader (Tecan, Switzerland). The luciferase activity value was calculated using the ratio of Ff/Rn.

### 4.11. Chromatin Immunoprecipitation (ChIP) Assay

The IPEC-J2 were treated with 1% formaldehyde on a shaker for crosslinking, then ended with 50 mM Glycine. In order to obtain chromatin fragments, the cells were lysed with a lysed buffer and sonicated with Bioruptor for 30 min below 4 °C. Then, the immunoprecipitation was done by incubating overnight at 7 °C with the Sp1 antibody (Proteintech, 21962-1-AP) or the control rabbit IgG antibody (DIA-AN, Q6005). Next, the crosslinking was reversed, and the DNA was enriched and purified using a FastPure Gel DNA Extraction Mini Kit (Vazyme Biotech Co., Ltd., Nanjing, China). The immunoprecipitated chromatin was quantified by qPCR using the primers listed in Table S10.

### 4.12. RNA-Seq and Bioinformatic Analysis

The total RNA was extracted from the *RIOK2* gene interference cell line (si-*RIOK2*, *n* = 4) and DON-treated *RIOK2* gene interference cell line (si-*RIOK2* + DON, *n* = 4). The RNA Nano 6000 Assay Kit of the Bioanalyzer 2100 system (Agilent Technologies, Palo Alto, CA, USA) was used to assess the RNA integrity. Using an Illumina Novaseq platform, the reads were generated from the library preparations, followed by the removal of reads containing an adapter, reads containing ploy-N, and low-quality reads from raw data. Then, quantification of the gene expression level and differential expression analysis were conducted. Genes with an adjusted *p*-value < 0.05 and |log_2_ (fold change)| > 1 were classified as differentially expressed. WGCNA (weighted correlation network analysis) was used to integrate the data obtained with the data of IPEC-J2 (J2, *n* = 4) and DON-treated IPEC-J2 (J2 + DON, *n* = 4) based on our previous study [17]. The GO and KEGG enrichment analyses were performed for the subsequent downstream analysis.

### 4.13. SNP Analysis and Construction of Vectors

In total, 300 Large White pigs were obtained from Changzhou Kangle Farming Co., Ltd. (Changzhou, China). About 1.0 g of ear tissue was collected from each individual and placed into a sterile tube. The total DNA was extracted from the samples using a DNA extraction kit (TIANGEN, Beijing, China). The concentration and purity of DNA were assessed using a NanoDrop 2000 spectrophotometer (Thermo Fisher Scientific, Wilmington, DE, USA). PCR primers (Table S11) were designed using Primer Premier 5.0 software according to the promoter sequence of the porcine *RIOK2* gene. The PCR assay was conducted in 50 µL volume containing 500 ng of the DNA template, 25 µL of 2 × Rapid Taq Master Mix, 20 pmol of each forward and reverse primer, and distilled water up to 50 µL. The reaction procedure was as follows: 95 °C for 3 min, 35 cycles of 95 °C for 15 s, 60 °C for 15 s, 72 °C for 30 s, and final extension at 72 °C for 5 min. The PCR products were sequenced by Sangon Biotech (Shanghai, China). The sequences were compared to each other using the BioEdit 7.2 software. Afterwards, the wild-type and mutational oligos of the *RIOK2* promoter region were constructed (Table S12) and were recombined into the pGL3-basic vector, followed by transformation into DH5α competent cells. After culturing on a solid medium containing ampicillin for 16 h, monoclonal colonies were picked, plasmids were extracted, and double digestion and sequencing were performed.

### 4.14. Statistical Analysis

Each experiment was replicated at least three times, and the results are presented as the mean ± standard deviation (SD). Unpaired *t*-test was used to perform statistical comparisons using GraphPad Prism 8.0 software (GraphPad, Inc., San Diego, CA, USA). The significance level was shown as follows: * *p* < 0.05; ** *p* < 0.01.

## Data Availability

The data presented in this study are available on request from the corresponding author.

## Supplementary Materials

The following supporting information can be downloaded at: https://www.mdpi.com/article/10.3390/ijms232112712/s1.
